# Impaired quality of life, but not cognition, is linked to a history of chronic hypercortisolism in patients with Cushing’s disease in remission

**DOI:** 10.3389/fendo.2022.934347

**Published:** 2022-08-08

**Authors:** Emilie Pupier, Alicia Santos, Nicole Etchamendy, Aurélie Lavielle, Amandine Ferriere, Aline Marighetto, Eugenia Resmini, Daniela Cota, Susan M. Webb, Antoine Tabarin

**Affiliations:** ^1^ Department of Endocrinology, Diabetes and Nutrition, CHU of Bordeaux and University of Bordeaux, Pessac, France; ^2^ Endocrinology Department, Centro de Investigación Biomédica en Red de Enfermedades Raras (CIBERER, Unidad 747) Instituto de Salud Carlos III (ISCIII), Barcelona, Spain; ^3^ Department Medicine, Research Center for Pituitary Diseases, Hospital Sant Pau, Institut d'Investigació Biomèdica (IIB)-Sant Pau, Universitat Autònoma de Barcelona (UAB), Barcelona, Spain; ^4^ Neurocentre Magendie, University of Bordeaux, Institut National de la Santé et de la Recherche Médicale (INSERM), Bordeaux, France

**Keywords:** Cushing’s disease, hypercortisolism, cognition, memory, quality of life

## Abstract

**Context:**

Impaired cognition and altered quality of life (QoL) may persist despite long-term remission of Cushing’s disease (CD). Persistent comorbidities and treatment modalities may account for cognitive impairments. Therefore, the role of hypercortisolism *per se* on cognitive sequelae remains debatable.

**Objective:**

To investigate whether memory and QoL are impaired after long-term remission of CD in patients with no confounding comorbidity.

**Design and Setting:**

Cross-sectional case-control study in two tertiary referral centers

**Patients:**

25 patients (44.5 ± 2.4 years) in remission from CD for 102.7 ± 19.3 Mo and 25 well-matched controls, without comorbidity or treatment liable to impair cognition.

**Main Outcome Measure(s):**

Hippocampus- and prefrontal cortex-dependent memory, including memory flexibility and working memory, were investigated using multiple tests including sensitive locally-developed computerized tasks. Depression and anxiety were evaluated with the MADRS and HADS questionnaires. QoL was evaluated with the SF-36 and CushingQoL questionnaires. The intensity of CD was assessed using mean urinary free cortisol and a score for clinical symptoms.

**Results:**

CD patients displayed similar performance to controls in all cognitive tests. In contrast, despite the absence of depression and a minimal residual clinical Cushing score, patients had worse QoL. Most of the SF36 subscales and the CushingQoL score were negatively associated only with the duration of exposure to hypercortisolism (p≤ 0.01 to 0.001).

**Conclusions:**

Persistent comorbidities can be a primary cause of long-lasting cognitive impairment and should be actively treated. Persistently altered QoL may reflect irreversible effects of hypercortisolism, highlighting the need to reduce its duration.

**Clinical Trial Registration number:**

https://clinicaltrials.gov, identifier NCT02603653

## Introduction

Chronic hypercortisolism significantly impacts brain function and morphology (1–5). Cognition is frequently negatively affected during Cushing’s disease (CD) including deficits in memory, verbal and visual learning (4, 6, 7). Imaging has revealed cerebral atrophy and functional changes in areas rich in glucocorticoid receptors, involved in processing of cognitive functions, such as the hippocampus, amygdala, and prefrontal cortex (1, 8). Remission of CD is usually associated with cognitive improvements (4, 6, 7, 9), but controversies persist as to the degree of recovery (4, 6, 7). Numerous factors may account for the heterogeneity between the results of studies. These include variations in recruitment, duration of hypercortisolism and time elapsed between remission of CS and cognitive evaluation, assuming that cognitive recovery may be delayed (2, 10, 11), discrepancies between cross-sectional and longitudinal studies, and differences in neuropsychological tests used (6, 7, 11, 12). Besides these caveats, variable memory recovery between individuals questions the specific role of hypercortisolism and suggests that different causes, directly or indirectly linked to hypercortisolism, may be involved in persistent alterations (12, 13). Indeed, patients may have confounding factors impairing cognition and, more specifically, memory. These include advanced age (14), obesity, poorly-controlled diabetes (15, 16), cerebral vascular disease and poorly controlled hypertension (17), psychopathology and depression including psychotropic treatments (18), excessive hydrocortisone replacement, imperfect hormonal replacement for hypothyroidism and untreated GH deficiency (19–21). Among the treatment modalities, pituitary radiotherapy may be associated to cognitive deficits (22, 23). A number of these putative interfering factors were not controlled or specified in prior publications. Therefore, the specific long-term consequences of hypercortisolism after its cessation on cognition remain debatable. In addition, identifying the association of confounding factors with persistent cognitive deficits is clinically relevant, because they may lead, along with somatic sequelae, to a lasting deterioration in quality of life (QoL) (2–4, 6, 7, 24, 25). To address this, we investigated memory function with multiple tests, including sensitive computerized tests for declarative and working memory, and QoL in patients with long-term remission of CD, with no confounding comorbidity.

## Patients and methods

### Patients

Clinical charts of 192 patients with cured CD and followed for > 1 year were reviewed in two expert centers (Endocrinology departments of Haut Leveque Hospital, Bordeaux, France and Hospital Sant Pau, Barcelona, Spain). CD was confirmed on histopathological analysis, remission of CD following transsphenoidal surgery, and the results of bilateral inferior petrosal sinus sampling (BIPSS). Patients were selected using two main criteria: 1) being in definitive remission following surgery for at least one year and 2) the absence of confounding factors or comorbidity that may alter cognitive functions.

Remission was ascertained during the 3 months preceding cognitive evaluation and was defined by persistent adrenal insufficiency or normal 24h urinary free cortisol excretion (UFC) associated with serum cortisol < 50 nmol/L following 1 mg overnight dexamethasone suppression test. Patients with adrenal insufficiency had to receive hydrocortisone supplementation at doses ≤ 15 mg/m^2^. Conditions known to interfere with cognitive function (considered exclusion criteria) included: age > 60 years, remission of CD induced by drugs, previous pituitary radiotherapy, BMI > 30 kg/m², drugs- and alcohol-abuse (past and present), untreated GH-deficiency or hypothyroidism, uncontrolled diabetes (HbA1c > 7.5% or FBG > 1.40 g/L) or diabetes-induced organ damage, past-history of neurological/vascular disease including uncontrolled hypertension, intake of psychotropic drugs including sleeping tablets and intake of exogenous steroids. GH secretion was assessed using dynamic testing and was considered normal in presence of a GH peak > 3 ng/mL during insulin tolerance test and glucagon stimulation test or a GH peak > 8 ng/mL during the GHRH -arginine stimulation test.

### Control group

Each patient with CD recruited a control subject matched for age, gender, living area, socioeconomic status and educational level. Five educational levels were defined: 1: no school, 2: primary school, 3: 5-9 years at school, short secondary school, 4: more than 9 years at school, long secondary school, 5: more than 12 years, university level. Exclusion criteria for controls were identical to those of CD patients; thyroid function was evaluated, but GH secretion was not.

Informed consent was obtained from all participants and the study was approved by the ethical committees of both centers. The study was registered under Clinical Trials (ClinicalTrials.gov Identifier: NCT02603653).

### Methods

All evaluations for the patients and the controls were performed at the same time during one day.

### Cognitive evaluation

Cognitive and psychological evaluation was performed by a dedicated psychologist to explore memory (hippocampal- and frontal- dependent forms), fluency, mood (including depression and anxiety); and QoL. The neuropsychological outcomes were explored using: 1) The Rey Auditory Verbal Learning Test (RAVLT) to evaluate verbal episodic memory (26), 2) the Isaac’ Set tests assessing phonemic and semantic verbal fluency abilities and speed of verbal production (27), 3) two subtests of the Wechsler Memory Scale, 4th Edition that evaluates visual and visuo-spatial working memory: the spatial addition task and the symbol span task (28), 4) the Rey-Osterrieth Complex Figure (ROCF) test dedicated to the evaluation visuospatial constructional ability and visual memory (29). Only the delayed recall was performed in this test.

In addition, patients underwent two locally developed computerized maze memory tests assessing cardinal features of declarative memory and working memory such as flexibility of declarative memory; and organization of information in memory and sensibility to interference (30–32) (see **Supplemental Data**).

### Evaluation of depression and anxiety

Depression and anxiety were assessed using the Depression Rating Scale (MADRS) questionnaire and the Hospital Anxiety and Depression Scale (HADS) (33, 34).

### Evaluation of QoL

The generic Short Form 36 (SF-36) questionnaire (35) was used in both patients and controls. The specific CushingQoL questionnaire was also used in patients (36).

### Evaluation of Cushing’s disease

We used an arbitrarily defined score for symptoms of hypercortisolism to evaluate clinical intensity of CD. The score was calculated following clinical examination at the time of cognitive evaluation and retrospectively calculated from medical reports at diagnosis. A score of 1 was recorded for the presence of any symptom like hirsutism, menstrual irregularities, buffalo neck, facial plethora and central obesity, diabetes, dyslipidemia, hypertension and cognitive or psychopathological complaints. A score of 2 was recorded for the presence of more specific symptoms such as large purple striae, proximal muscle weakness, spontaneous ecchymosis and glucocorticoid-induced osteoporosis. The maximum score was 20. The biological intensity of CD at diagnosis was calculated using the mean results of 2-3measurements of 24h UFC expressed relative to the upper normal range of the assay (x ULN).

The apparent duration of hypercortisolism was calculated retrospectively from the time between onset of symptoms according to the patient interview, and the date of remission of hypercortisolism.

### Statistical analysis

A power analysis was performed: according to a hypothesis that the percentage of correct answers to the Bordeaux maze test would be 80% in controls, with a standard deviation of 13% and 60% with a standard deviation of 23% in CD patients, 25 patients per group were necessary to have a power of 80% associated with a type 1 risk of 5%. Cognitive performance parameters recorded in each task and scores recorded in the different scales assessing QoL, anxiety and depression were analyzed with ANOVAs to assess potential effect of group factor. Comparison of data between patients with recovery of the HPA axis and those with persistent corticotropic insufficiency were analyzed using the t-test or Mann-Whitney’s test according to the distribution of values. All results are expressed as mean ± SEM. Multiple correlation analysis were performed with Spearman test after Log transformation of data using GraphPad software (San Diego, CA). We also compared Z-scores (computed using log data for each participant taking into account the mean and SD of each measure) of SF36 items in controls versus patients. A negative z-score indicates that patients has lower quality of life than the corresponding normative population. The Bonferroni method was used to reduce the likelihood of type I error. Since we performed 4 different evaluations (cognitive, depression, anxiety, and quality of life), a p value ≤0.01 was considered as significant.

## Results

### Characteristics of patients and controls

**Figure 1:**
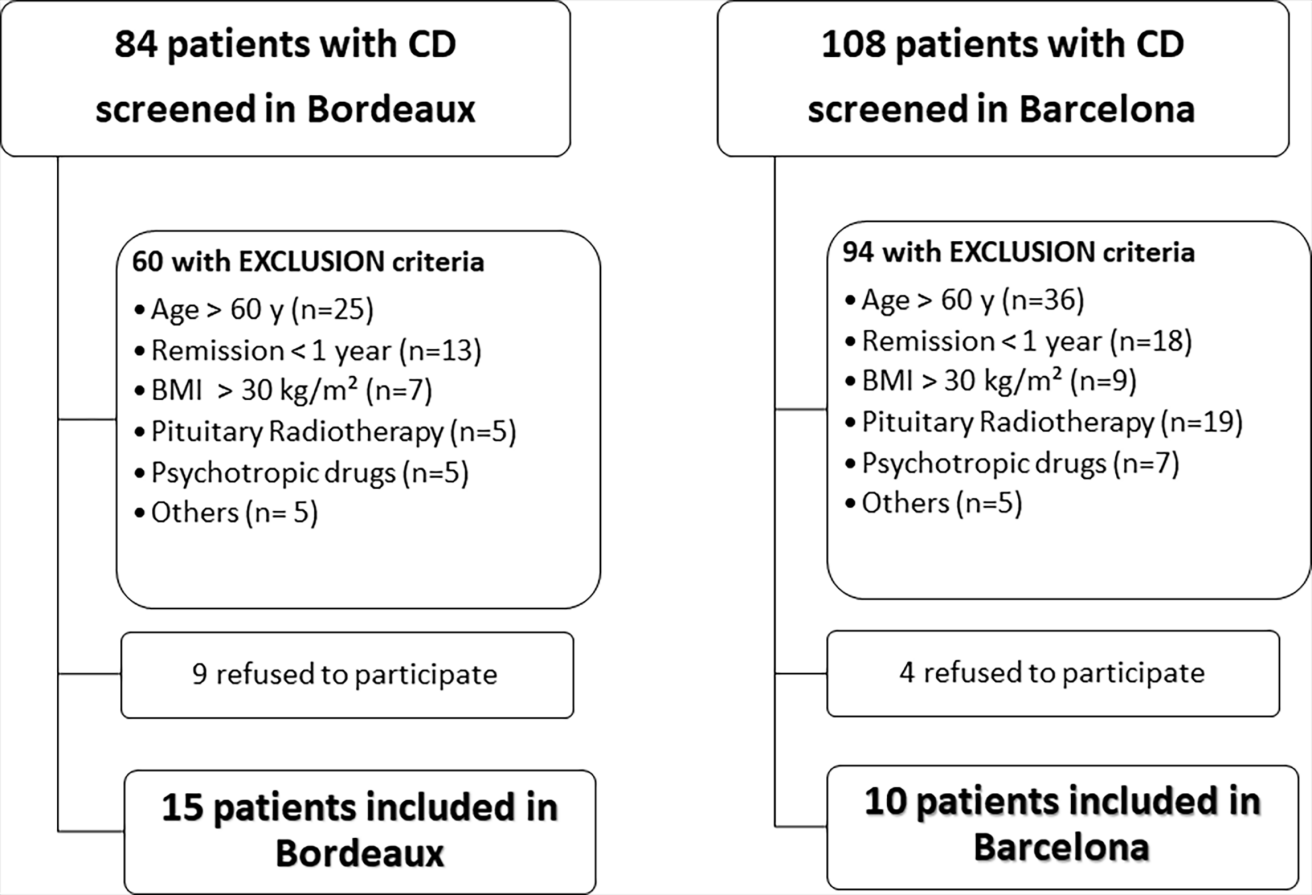
Flow chart of the recruitment of patients with Cushing’s disease (CD) in the two investigating centers.

Clinical files of 192 patients with CD were analyzed. Twenty-five (19 women and 6 men, aged 44.5 ± 2.4 y) in long-term remission of CD (102.7 ± 19.3 months; range =17-364) satisfied the inclusion criteria and accepted to participate (**Table 1** and **Figure 1**). Ten were recruited in Barcelona and 15 in Bordeaux. Twenty-two were cured following a single pituitary surgery. Among these, 13 patients had a normal biological evaluation of the HPA axis and 9 had persistent corticotropic insufficiency. Three underwent bilateral adrenalectomy following 1 (n=1) or 2 (n=2) unsuccessful pituitary surgical attempts. Hydrocortisone was given at 11.3 ± 1.9mg/m² dose divided in 2-3 daily intakes in 12 patients (e.g. 10 mg/day in 3, 11 to 20 mg/day in 5 and 21 to 30 mg/day in 4.

**Table 1:** Characteristics of participants in the Memocush study.

	Patients (n=25)	Controls (n=25)
Age (years)	44.5 +/- 2.4	44.3 +/- 2.3
Women (%)	76	76
Level of education (from 1 to 5)	Level 4 (n=8) & Level 5 (n=17)	Level 4 (n=7) & Level 5 (n=18)
BMI (kg/m^2^)	24.2 +/- 0.6	23.1 +/- 0.6
Treated hypertension (N)	5	0
Systolic blood pressure (mm Hg)	123.2 +/- 3.2	116.6 +/- 2.6
Diastolic blood pressure (mm Hg)	74.4 +/- 2.0	71.3 +/- 2.3
Diabetes mellitus (N)	1	0
Fasting glycemia (mmol/l)	4.9 +/- 0.1	5.1 +/- 0.1
HbA1c (%)	5.4 +/- 0.1	5.4 +/- 0.1
Hydrocortisone treatment (Number of patients)	12	0
Hydrocortisone total dose (mg/m² per day)	11.3 +/- 1.9	NA
Treated Hypothyroidism (Number of patients)	5	1
Treated GH deficiency (Number of patients)	2	0
Clinical score at diagnosis (/20)	12.6 +/- 0.7	NA
Clinical score at evaluation (/20)	2.3 +/- 0.5	NA

No significant difference was found in the non-specific parameters between patients with a history of Cushing’s disease and their matched controls.

Thirteen patients had a BMI ≥ 25 and < 30 kg/m^2^. Five were supplemented with levothyroxine and 2 with recombinant GH. Blood pressure was controlled pharmacologically in 5 hypertensive patients (≤135/85 mmHg); only one patient was treated with antidiabetic hypoglycemic oral drugs (HbA1C = 6.8%).

Mean UFC at diagnosis was 4.4 ± 0.6 x ULN (range 1.5 – 12.4). An improvement in the clinical score was observed between diagnosis and the time of evaluation (12.6 ± 0.7 vs 2.2 ± 0.5, respectively; p < 0.0001). Clinical scores at diagnosis and at the time of evaluation were similar between patients with and without adrenal insufficiency (13.5 ± 1.0 and 11.7 ± 0.9; p=0.2 and 2.3 ± 0.8 vs 2.2 ± 0.5, p = 0.91, respectively).

Twenty-five subjects (19 women and 6 men, aged 44.3 ± 2.3 y), were recruited as controls. CD patients and controls had similar characteristics (**Table 1**). Apart from one individual receiving adequate levothyroxine supplementation for hypothyroidism, no control subject had a history of endocrine disease.no control subject had a history of endocrine disease.

### CD patients in long-term remission have preserved cognitive performance

CD patients in long-term remission displayed similar performance to controls in all cognitive tests. Regarding declarative memory, total words recalled in the RAVLT test was not different between control and CD groups [group effect: F (1,48)=1.81; p=0.18] and CD patients in remission performed as controls in all RAVLT subtests [i.e. delayed recall: F (1,48)=1.7; p=0.19, for all the subtests, mean and p value in **Table 2**]. In the Bordeaux Maze declarative memory test, patients learned accurately the initial 6 pairs, needing similar number of training sessions as controls [group effect: F (1,48)=1.7; p=0.19]. When considering the evolution of performance during the last 6 trials preceding achievement of criterion performance, CD patients progressively reached a mean percent of correct choices (96%) close to that of controls (98%), with no difference between both groups (group effect: F (1,48)=1.8; p=0.31; interaction trials X group: F (5,240)=1.2; p=0.19). When submitted to flexibility probes, CD patients accurately expressed their memories of previously acquired information similarly to controls [group effect: F (1,48)=0.7; p=0.39]. 6/25 CD patients displayed performance in the flexibility probes below or equal to 62.5% of correct responses, similar to that observed in the controls.

**Table 2:** Mean responses during various cognitive tests.

	Controls		CD		p value
**List A-1**	7.1 ± 0.5	(1/25)	6.4 ± 0.5	(5/25)	p=0.24
**List A-2**	10.6 ± 0.6	(1/25)	9.7 ± 0.5	(1/25)	p=0.26
**List A-3**	12.0 ± 0.6	(1/25)	11.0 ± 0.4	(1/25)	p=0.16
**List A-4**	12.7 ± 0.4	(1/25)	12.2 ± 0.4	(1/25)	p=0.4
**List A-5**	13.2 ± 0.3	(1/25)	12.7 ± 0.4	(1/25)	p=0.31
**List A-6-Retention**	12.0 ± 0.5	(1/25)	10.7 ± 0.6	(1/25)	p=0.09
**Delayed A**	12.2 ± 0.5	(1/25)	11.2 ± 0.5	(1/25)	p=0.19
**P**	26.0 ± 1.3	(0/25)	26.6 ± 1.3	(0/25)	p=0.76
**R**	22.5 ± 1.3	(2/25)	20.5 ± 1.3	(1/25)	p=0.28
**V**	16.5 ± 0.9	(1/25)	17.2 ± 1.0	(0/25)	p=0.59
**Animal**	33.5 ± 1.5	(2/25)	33.6 ± 1.6	(0/25)	p=0.94
**Fruit**	17.0 ± 1.0	(9/25)	16.5 ± 1.0	(7/25)	p=0.74
**Furniture**	21.7 ± 1.0	(0/25)	21.4 ± 0.7	(0/25)	p=0.27
**Spatial Addition**	14.1 ± 0.8	(4/25)	13.7 ± 0.7	(4/25)	p=0.69
**Symbol**	25.2 ± 1.3	(3/25)	26.2 ± 1.6	(4/25)	p=0.63
**ROCF Copy**	55.6 ± 3.6	(0/25)	54.6 ± 3.6	(0/25)	p=0.87
**ROCF delayed**	33.1 ± 2.6	(1/25)	31.4 ± 2.6	(1/25)	p=0.1

Correct responses in the successive free recall subtests of the RAVLT (26); words produced for each letter or category subtest of the Isaac Set Test (27), performance in the spatial addition test, the symbol test and the ROCF test (29). For each parameter, the number of deficient participants according to the published normative value is specified and the p value indicates the effect of group. CD: patients with a history of Cushing’s disease.

Regarding working memory, symbol span score was not different between CD patients and controls [group effect: F (1,48)=0.2; p=0.63]. In the spatial addition subtest, CD patients in remission performed similarly to controls [group effect: F (1,48)=0.2; p=0.69]. In the Bordeaux Working memory test, the total mean correct performance of the CD group was above chance level and not different from controls [group effect: F (1,48)=1.9; p=0.16].

Regarding verbal fluency, CD patients in long-term remission performed similarly to controls both in the semantic subtest [group effect: F (1,48)=0.2; p=0.67] and in the phonetic subtest [group effect: F (1,48)=0.03; p=0.85].

The ROCF scores were all found to be within acceptable limits. Performance on the ROCF copy or the ROCF recall test were not different between both groups [copy: F (1,48)=0.03, p=0.87; recall: F (1,48)=0.09, p=0.76]

According to published normative values for RAVLT-, Isaacs-, spatial addition- and symbol tests, the proportion of patients with abnormal responses was similar in CD and control groups.

Patients with normal HPA axis function performed better than patients requiring hydrocortisone supplementation only during the Isaac lexical test (24.0 ± 1.2 vs 18.6 ± 1.1; p < 0.005).

### CD patients in long-term remission have substantially impaired QoL

CD patients scored worse than controls in most subscales of the SF-36 questionnaire taken independently with differences reaching statistical significance for physical functioning, role-physical, general health and vitally:[physical functioning: F (1,48)=15.6; p<0.005; role-physical: F (1,48)=9.2; p<0.005; general health: F (1,48)=10.2 p<0.005; vitality: F (1,48)=6.3; p<0.01; social functioning: F (1,48)=5.1 p<0.05; role-emotional: F (1,48)=3.5; p=0.06; bodily pain: F (1,48)=1.7; p=0.19; mental health: F (1,48)=3.04; p=0.08] (**Table 3**). Z scores of SF36 (especially physical functioning, general Health and vitality) were also significantly lower in CD patients than in controls (**Supplementary Table 1**).

**Table 3:** Mean score in each subscale of the SF-36 for participants.

	SF36-PF	SF36-RP	SF36-BP	SF36-GH	SF36-VIT	SF36-SF	SF36-RE	SF36-MH
Controls	94.8 ± 1.6 2/25	87.7 ± 5.1 2/25	86.1 ± 3.8 1/25	74.8 ± 2.7 1/25	70.0 ± 3.7 3/25	92.4 ± 3.8 3/25	90.7 ± 4.4 1/25	81.2 ± 2.9 1/25
CD	78.9 ± 3.7 10/25	58.9 ± 7.1 8/25	78.5 ± 4.4 1/25	57.0 ± 4.8 7/25	54.2 ± 5.1 12/25	77.8 ± 5.5 6/25	74.9 ± 7.2 5/25	72.7 ± 3.8 3/25
*p* value	*p*=0.0003	*p*=0.0039	*p*=0.19	*p*=0.0024	*p*=0.015	*p*=0.03	*p*=0.068	*p*=0.087

CD, patients with a history of Cushing’s disease. For each parameter; the number of deficient participants according to the published normative value is specified and the p value indicates the effect of group. PF, physical functioning; RP, Role Physical; BP, Bodily Pain; GH, General Health; VIT, Vitality; SF, Social Function; RE, Role Emotional; MH, Mental Health.

CD patients scored worse than controls in the depression rating scale [MADRS: F (1,48)=3.8; p = 0.01]. 10/25 CD patients displayed mild (8/25) or moderate (2/25) depression as opposed to 12% 3/25 of the controls (p=0.04). These mild alterations were not apparent in the depression subscale of the HADS questionnaire [F (1,48)=0.51; p=0.47]. Similarly, no difference was found between patients and controls in the anxiety subscale of the HADS questionnaire [F (1,48)=2.46; p=0.12] (**Table 4**). The CushingQoL global score was 62.6 ± 3.9.

**Table 4:** Mean score in each subscale of the Hospital Anxiety and Depression Scale (HADS) and the Depression Rating Scale (MADRS) for each group of participants.

	HADS Depression	HADS Anxiety	MADRS
Controls	3.1 ± 0.7 Doubtful cases : 1/25 Certain cases : 1/25	6.6 ± 0.7 Doubtful cases : 1/25 Certain cases : 4/25	5.0 ± 1.4 Mild depression : 2/25 Moderate depression : 1/25
CD	3.8 ± 0.6 Doubtful cases : 3/25 Certain cases : 0/25	7.4 ± 0.7 Doubtful cases : 3/25 Certain cases : 7/25	9.8 ± 1.1 Mild depression : 8/35 Moderate depression : 2/25
*p* value	*p*=0.47	*p*=0.12	*p*=0.04

CD, patients with a history of Cushing’s disease. For each test, the number of participants with abnormal score is specified according to normative values and the p value indicates the effect of group.

There was no significant difference in the results of tests between patients requiring hydrocortisone supplementation and patients with a normal HPA axis. Only a nominal association was found for the general health perception of the SF-36 questionnaire (47.3 ± 7.8 vs 66.1 ± 4.9 for patients taking hydrocortisone and patients with normal HPA axis function respectively; p = 0.05).

### Worse QoL is correlated to the duration of exposure to cortisol excess

Biological intensity of hypercortisolism was not correlated to the clinical score at diagnosis, nor to the clinical score at the time of evaluation (r=0.39; p=0.10 and r=0.32; p=0.19, respectively). None of the QoL, cognition and anxiety/depression evaluations including all SF-36 subscales correlated with the biological intensity of hypercortisolism and clinical score at diagnosis (p = 0.16 to 0.99). In contrast, the general health, vitality, role physical and physical functioning subscales of the SF-36, as well as CushingQoL were significantly and nominally negatively associated with the duration of exposure to hypercortisolism: r=-0.54, p= 0.004; r=-0.61, p=0.001; r=-0.53, p=0.01; r=-0.49, p=0.02; r=-0.43, p=0.04; respectively. No parameter was associated with the time elapsed between remission and time of testing (p=0.19 to 0.85).

## Discussion

The main finding of our study is that, in the absence of obvious confounding comorbidity, adult patients younger than 60 years in surgical remission of CD displayed no long-term memory impairment. These findings were obtained after multiple tests for memory evaluation, including the Bordeaux Maze relational memory test that has been shown to be exquisitely sensitive to mild changes in declarative memory, as occurs with ageing (30, 31, 37, 38). In contrast, and in the absence of obvious psychopathology and major physical sequelae, patients displayed impaired QoL, the intensity of which was proportional to the duration of active hypercortisolism.

Studies evaluating patients in remission of CD show variable improvement in cognitive domains and a recent publication pinpointed that the scarcity of data did not allow metanalysis of studies for memory (6). We hypothesized that cortisol-related comorbidities and treatments of CD may account for persistent memory deficits and impaired QoL. This study was therefore carried out in an attempt to discriminate the respective impact of a direct effect of hypercortisolism on the brain versus that of other causes, directly or indirectly linked to hypercortisolism in persistent memory impairment and altered QoL.

Thus, we strictly excluded putative confounding factors which may explain the discrepancy with more pessimistic studies on the recovery of cognitive functions (9–13, 39–43). For example, while most investigators used an age limit of 70, we decided, based on studies devoted to the decline in memory performance with age (31, 37, 44), to use an age limit of 60 years. Several studies included patients treated for depression. However, both depression and long-term intake of antidepressant drugs can induce cognitive impairments (18, 45). Similarly, the use of any psychotropic drug including sleeping pills, described in almost 1/3 of patients with a history of CD (46), was an exclusion criterion. No major psychological disturbance was found in our patients. A trends towards increased prevalence of depression was found using the MADRS, but depression was rated as mild and patients did not differ from controls during the HADS screening questionnaire for depression and anxiety. Accordingly, the mental health dimension of the SF-36 questionnaire was similar to that of controls. Given the debate on the consequences of brain irradiation, patients after pituitary radiotherapy were excluded (9, 22). Hydrocortisone replacement dosage and GH deficiency were also controlled to avoid interference between imperfect hormonal replacement and cognitive performance (19–21). A delay between remission of CD and recovery of cognitive impairments has also been suggested (2, 10). The longer duration of remission (mean = 8.6 years) before cognitive evaluation in our study as compared to several published studies may partly account for our findings. Interestingly, one of the largest published studies with the longest duration of remission, that also considered a number of confounding comorbidities for selecting patients, only found subtle long-term residual alterations in cognitive function and memory (9). The mild differences with the results of our study, in which several similar cognitive tests were used may also be related to some residual confounders. For instance, our patients were younger (44 ± 2 years vs 52 ± 1 years; P = 0.008) and had a higher educational level, two parameters associated with the outcome of most cognitive tests (9, 13, 43). In addition, 27% of the Dutch patients underwent pituitary radiotherapy, a treatment modality negatively associated with memory and executive functioning (9).

In accordance with previous studies using generic and disease-specific questionnaires (2, 6, 7, 24, 25, 47), CD patients in remission demonstrated persistent worse QoL, with a similar CushingQoL score to those previously published (48). Depression and physical sequelae are major determinants of altered QoL (48). Therefore, an intriguing finding of our study is the contrast between persistently altered QoL on the one hand, and lack of memory impairment, evident psychopathology and major residual somatic comorbidity as illustrated by the minimal residual clinical score on the other hand. These differences between perception and objective findings are reminiscent of the description of an increased negative illness perception in CD (49). Subtle psychopathological alterations and unconscious fears related to the mental experience of hypercortisolism that cannot be detected by questionnaires cannot be excluded. Close links between glucocorticoids and addiction have been described in animal models including mediation of addictive properties of drugs by glucocorticoids (50, 51). The positive reinforcing effects of sustained hypercortisolism may determine reward-related psychopathologies (51) and, due to their psychostimulant properties, corticosteroids have been labelled as drugs of addiction (52). The negative emotional state and symptoms of drug abuse withdrawal (53) are also observed after remission of hypercortisolism, at least in the short-term (54). One could hypothesize that prolonged impaired QoL may share common mechanisms with those observed following withdrawal from drug abuse. In this conceptual framework, it is worth mentioning that improvements in QoL in this last situation can be delayed for decades, with lower indices of well-being over time in women with a history of psychostimulant abuse as compared to alcohol and cannabis abuse (55). Improvement in psychological care of patients with a history of CD may also benefit from neuroendocrine research in the field of addiction.

Importantly, most of the SF-36 subscales and the CushingQoL score were negatively associated with the calculated duration of exposure to hypercortisolism, but not with the duration of remission. This has also been described in a retrospective survey (47) and is reminiscent of studies showing that the persistence of somatic and psychological cortisol-related comorbidities correlates with the delay to diagnosis (56).

Our study has several limitations. Being cross-sectional, it does not allow comparison with alterations observed during the active phase of CD or concluding that cognitive dysfunction in CD is reversible. However, a cross-sectional study avoids learning the cognitive tests that occurs with their repetition and may result in an apparent improvement of performances. In addition, our study probably escapes the selection bias of longitudinal studies with high losses at follow-up, that may select patients who perform worse (6, 7). Dissociations in the recovery between various cognitive domains have been reported (10, 11) and we cannot exclude the persistence of other less frequently observed cognitive deficits, not explored within our protocol.

The number of patients is relatively small, a problem practically unavoidable in rare diseases, especially if followed up over years, but compares well to most series related to memory performance published to date (6, 10, 12, 39, 41–43). As potent confounders are very common in patients with a history of CD, an important proportion of patients were excluded. Although we agree that this selection excludes the most common situation in which patients have persistent comorbidities, it was mandatory to delineate the specific role of past hypercortisolism and that of persistent cortisol-dependent or non-cortisol-dependent comorbidities on cognition and QoL. However, the comparison of parameters between patients with adrenal insufficiency and those with normal HPA axis function may lack statistical power given the small number of patients.

Among the methodological merits of our study, the careful matching of patients with controls is worth mentioning, a condition that likely resulted in a higher accuracy than studies involving population-based cohorts. In addition, the selection of controls by patients enabled a perfect match for socioeconomic status, which may be a determinant of the outcomes of the questionnaires, along with age, gender, and education level.

In conclusion, our study challenges the concept of irreversible memory impairment due to a specific and direct effect of hypercortisolism in patients below the age of 60. Although an increased vulnerability of the brain to cortisol excess in aged people cannot be excluded, our results suggest that various persisting co-morbidities of CD may be responsible for long-lasting impaired memory and should therefore be actively sought and adequately treated by expert specialists (46). The contrast between lasting impairment of QoL on the one hand and absence of major physical, psychological and cognitive sequelae on the other hand, may reflect irreversible consequences of cortisol excess. The association of negative health perception and impaired QoL with the duration of cortisol excess reinforces the importance of shortening this exposure, especially following diagnosis, to reduce the long-term burden of CD.

## Data availability statement

The raw data supporting the conclusions of this article will be made available by the authors, without undue reservation.

## Ethics statement

The studies involving human participants were reviewed and approved by CPP Sud-Ouest et Outre-Mer III and ethical committee of hospital de la Santa creu I Sant-Pau. The patients/participants provided their written informed consent to participate in this study.

## Author contributions

AS, NE, AM, DC, and AT conceptualized the study. NE and AM developed the Bordeaux maze investigations. EP, AL, and AS performed the investigations and collected the data. NE, EP, and AT analyzed the data. AS, ER, DC, SW, and AT wrote the manuscript. AT takes responsibility for the integrity of the data analysis AF performed the investigations and analyzed the data. All authors contributed to the article and approved the submitted version.

## Funding

This work was supported entirely with institutional funds and sponsored by Institut National de la Santé et de la Recherche Médicale (INSERM), France.

## Acknowledgments

We thank Prof. K Mohammedi for his assistance in the statistical analyses.

## Conflict of interest

The authors declare that the research was conducted in the absence of any commercial or financial relationships that could be construed as a potential conflict of interest.

## Publisher’s note

All claims expressed in this article are solely those of the authors and do not necessarily represent those of their affiliated organizations, or those of the publisher, the editors and the reviewers. Any product that may be evaluated in this article, or claim that may be made by its manufacturer, is not guaranteed or endorsed by the publisher.

## Supplementary material

Supplementary Data Sheet 1 Computerized maze memory tests assessing cardinal features of declarative memory and working memory.

Supplemental Table 1 Z-scores of each SF36 subscale in controls and patients with Cushing’s disease in remission Z-scores of log data were computed for each participant taking into account the mean and SD of log SF36 measures. A negative z-score indicates that the population has lower quality of life than the corresponding normative population.
