# Supplementary material for: Impaired quality of life, but not cognition, is linked to a history of chronic hypercortisolism in patients with Cushing’s disease in remission
Source: Front Endocrinol (Lausanne). 2022 Aug 8;13:934347. doi: 10.3389/fendo.2022.934347 (PMC9393704; doi:10.3389/fendo.2022.934347)
Supplement: Supplementary Data Sheet 1 — Computerized maze memory tests assessing cardinal features of declarative memory and working memory. [file DataSheet_1.docx]

**Supplemental data**

**Impaired quality of life, but not cognition, is linked to a history of chronic hypercortisolism in patients with Cushing's disease in remission by Emilie Pupier et al.**

**Supplemental data 1** **Computerized maze memory tests tests assessing cardinal features of declarative memory and working memory**

The first computerized test (OptoPath; ANR-10-EQX-008-1; Imetronic, Pessac, France) explores one fundamental property of declarative memory that is its flexible expression. Memory flexibility can be exemplified as the capability to compare separately acquired pieces of information in order to make a choice decision in a novel situation. The virtual radial maze 2-stages spatial discrimination task was specifically designed and automatized to assess this capability. Briefly, in the acquisition phase of the task, each subject was faced with successive presentations of six pairs of adjacent arms and required to visit one of the two arms during each trial. In each presentation, one arm always contained a reward (virtual coin) at its end, while the other arm never contained any reward. Training continued until reaching the acquisition criterion (when the number of incorrect choices of the nonrewarded arm was less than 2 over 12 consecutive trials), and a minimum of 6 trials or maximum of 20 trials were performed. Once the acquisition criterion was reached, the subject was then subjected to the probe test in which nothing was modified except that the arms were now presented in novel pairings (recombination of previous pairs) to assess the flexibility of memory expression. Typically, subjects with hippocampal dysfunction are able to learn the initial pairs, but are unable to express their knowledge in a flexible way (34,35).

The Bordeaux Maze Working Memory (OptoPath; ANR-10-EQX-008-1; Imetronic, Pessac, France) test assesses organization of information in memory and sensibility to interference. The structure of this computerized test is similar to that of the declarative memory task. The major difference is that the position of the reward within each pair was never the same from one trial to another following the rule of alternation: at each new presentation of a given pair, the path containing the reward was the one that the subject had not visited on the previous trial. Each subject was faced with successive presentations of 4 pairs and required to visit one of the two arms of each trial. To find the reward, the subject had to memorize the path that was visited at each presentation to alternate the next choice according to the choice made in the preceding trial with the same pair. Overall difficulty of the task varies regarding retention and interference management (organization demand) as a function of interfering pairs. This test has been demonstrated to diagnose an increased sensitivity to interference linked to frontal dysfunction related to aging in control populations (34,35).
