# Supplementary material for: Impaired quality of life, but not cognition, is linked to a history of chronic hypercortisolism in patients with Cushing’s disease in remission
Source: Front Endocrinol (Lausanne). 2022 Aug 8;13:934347. doi: 10.3389/fendo.2022.934347 (PMC9393704; doi:10.3389/fendo.2022.934347)
Supplement: Supplemental Table 1 — Z-scores of each SF36 subscale in controls and patients with Cushing’s disease in remission Z-scores of log data were computed for each participant taking into account the mean and SD of log SF36 measures. A negative z-score indicates that the population has lower quality of life than the corresponding normative population. [file Table_1.docx]

| **SF36 items** | **Controls** | **Cushing’s disease** | **P-values** |
| --- | --- | --- | --- |
| **Physical functioning** | 0.03 (0.03) | -0.04 (0.09) | 0.0004 |
| **Role Physical** | 0.05 (0.08) | -0.03 (0.14) | 0.03 |
| **Bodily Pain** | 0.002 (0.11) | -0.03 (0.12) | 0.28 |
| **General Health** | 0.03 (0.07) | -0.08 (0.17) | 0.002 |
| **Vitality** | 0.05 (0.05) | -0.09 (0.19) | 0.001 |
| **Social Function** | 0.01 (0.13) | -0.06 (0.15) | 0.10 |
| **Role Emotional** | 0.04 (0.04) | -0.01 (0.13) | 0.09 |
| **Mental Health** | 0.009 (0.09) | -0.03 (0.11) | 0.14 |

**Supplemental Table 1. Z-scores of each SF36 subscale in controls and patients with Cushing’s disease in remission**

Z-scores of log data were computed for each participant taking into account the mean and SD of log SF36 measures. A negative z-score indicates that the population has lower quality of life than the corresponding normative population.
